# Case Report: Theory of Mind and Figurative Language in a Child With Agenesis of the Corpus Callosum

**DOI:** 10.3389/fpsyg.2020.596804

**Published:** 2021-02-10

**Authors:** Sergio Melogno, Maria Antonietta Pinto, Teresa Gloria Scalisi, Fausto Badolato, Pasquale Parisi

**Affiliations:** ^1^Department of Developmental and Social Psychology, Sapienza University of Rome, Rome, Italy; ^2^Department of Neuroscience, Mental Health and Sensory Organs, Sapienza University of Rome, Rome, Italy; ^3^Faculty of Psychology, University Niccolò Cusano, Rome, Italy

**Keywords:** cognitive profile, theory of mind, figurative language, child, agenesis of the corpus callosum

## Abstract

In this case report, we studied Theory of Mind (ToM) and figurative language comprehension in a 7.2-year-old child, conventionally named RJ, with isolated and complete agenesis of the corpus callosum (ACC), a rare malformation due to the absence of the corpus callosum, the major tract connecting the two brain hemispheres. To study ToM, which is the capability to infer the other’s mental states, we used the classical false belief tasks, and to study figurative language, i.e., those linguistic usages involving non-literal meanings, we used tasks assessing metaphor and idiom comprehension. RJ’s intellectual level and his phonological, lexical, and grammatical abilities were all adequate. In both the ToM false belief tasks and novel sensory metaphor comprehension, RJ showed a delay of 3 years and a significant gap compared to a typically developing control group, while in idioms, his performance was at the border of average. These outcomes suggest that RJ has a specific pragmatic difficulty in all tasks where he must interpret the other’s communicative intention, as in ToM tasks and novel sensory metaphor comprehension. The outcomes also open up interesting insights into the relationships between ToM and figurative language in children with isolated and complete ACC.

## Introduction

The corpus callosum is the largest bundle of fibers connecting the cerebral hemispheres. The agenesis of the corpus callosum (ACC) is a rare malformation that occurs in 1:4,000 live births in which the approximately 200 million axons of the corpus callosum (Glass et al., 2008) are either absent or fail to migrate toward the opposite hemisphere (Bedeschi et al., 2006; Lund et al., 2016). Sometimes, this absence is complete, while in most cases, the anterior commissure is still present, which is labeled as partial ACC. Generally, the etiology cannot be identified, which corresponds to isolated or non-syndromic ACC, while in 30–45% of patients, a specific cause, either chromosomic anomalies or genetic syndromes (10% and 20–35%, respectively) can be detected (Bedeschi et al., 2006; Glass et al., 2008).

A recent meta-analysis (D’Antonio et al., 2016) has shown that individuals with isolated ACC (1.8 per 10.000) do not have additional syndromes nor other brain pathologies, but their cognitive and behavioral profiles are extremely heterogeneous. Intellectual quotient (IQ) can range from adequate to markedly delayed (Paul et al., 2007). Three core aspects have been identified in the neuropsychological profiles of individuals with an IQ above 80: diminished interhemispheric integration of sensory–motor information, slowed cognitive processing, and difficulty with complex reasoning and problem solving (Brown and Paul, 2019). This difficulty is also a function of age and task complexity (Siffredi et al., 2018; Brown and Paul, 2019). In the social interaction area (Badaruddin et al., 2007), particular difficulties have been found in identifying others’ mental states, which constitutes the core of the construct of the Theory of Mind (ToM) (Premack and Woodruff, 1978). These difficulties in the social area stimulated the researchers to compare the symptomatology of individuals with autism spectrum disorder (ASD, henceforth) with that of individuals with ACC, and in some cases, an overlapping between the characteristics of the two conditions was found (Booth et al., 2011; Paul et al., 2014). In particular, when comparing 26 adults with ACC with 28 adults with ASD (IQ > 78) Paul et al. (2014) found that eight out of 26 of the individuals with ACC showed symptoms that were compatible with a classification of autism based on ADOS 2 (Lord et al., 2012), but only three of them had a clinical history compatible with autistic development. To account for the characteristics of that subgroup, the authors thought that those individuals had a different developmental trajectory, with a delayed onset of autistic social symptoms and an early onset of restricted, repetitive, and stereotyped behaviors.

Regarding ToM, in the last decades, this construct has been explored more in depth from both the developmental (typical and atypical conditions) and neural point of view in terms of brain basis (Baron-Cohen et al., 2013). On the one hand, it has been clarified that ToM pertains to the wider area of social cognition and, in turn, is constituted by many components (Frith, 2008; Frith and Frith, 2008; Samson and Michel, 2013) such as high and low-level processes, as well as implicit and explicit processes. In some circumstances, implicit processes are sufficient to read the other’s mental states. This is the case, for instance, of appropriate reactions to facial expressions that communicate disgust or fear, or the capability to follow the gaze, imitate actions, and follow the other’s attentive focus (for a review, see Frith, 2008; Frith and Frith, 2008). On the other hand, in more complex circumstances as in the typical false belief tasks, inferential reasonings about mental states are required. Studies on patients with brain injuries highlighted the diversity of ToM components in false belief tasks: the capability to distinguish one’s own from the other’s mental state, to inhibit one’s own mental state in favor of the other’s, attentional monitoring to grasp relevant cues, working memory to maintain different mental states, and a set of concepts in long-term memory in order to plan an inferential reasoning (Samson and Michel, 2013).

The tasks devised to assess ToM reflect a further distinction between visual–perceptual processing of social and emotional cues, on the one hand, and verbal aspects, on the other. Regarding the capability to infer mental and emotional states from perceptual cues, the most widely used tasks request to read characters’ emotions from faces or eyes (Baron-Cohen et al., 2001) or prosodic cues in sentences (Golan et al., 2006). Classical instances of verbal tasks (Wimmer and Perner, 1983; Baron-Cohen et al., 1985) are the false belief tasks, first and second order. In typical development, the first-order false belief task is solved by age 4, while the second-order one is solved between ages 6 and 7 (Baron-Cohen et al., 1997).

A study (Bridgman et al., 2014) conducted on nine individuals with ACC (mean age range: 28 years) that explored facial scanning revealed less attention to the eye region compared to controls, which could contribute to a deficit in emotion recognition in the ACC group. Another study conducted by Symington et al. (2010) on 11 individuals with complete ACC (age range between 15 and 55 years) and normal intelligence revealed that in some ToM abilities, as assessed by Happé’s Theory of Mind Stories (Happé, 1994) and the Adult Faux Pas Test (Baron-Cohen et al., 1999), this sample was indistinguishable from a control group. On the other hand, there were deficiencies in the recognition of emotion and understanding paradoxical sarcasm, and a particular difficulty interpreting textual versus visual social cues as measured by the Thames Awareness of Social Inferential Test (TASIT; McDonald et al., 2003, 2006). This test requires to interpret videotaped social vignettes. The participants showed a series of weaknesses in integrating paralinguistic cues such as prosody and body language as well as textual cues that allow to understand non-literal language. Overall, these results seem to reflect a general bias toward literal interpretation.

Currently, very few studies have explored ToM in children with ACC. Labadi and Beke (2017) studied children with ACC aged 6–8 (16 with complete and two with partial ACC), compared to typically developing peers matched for IQ, age, gender, and education. The authors used the first- and second-order false belief tasks, and a mental and emotional state recognition task. The outcomes showed that, compared to the controls, children with ACC had mild difficulties in emotion recognition and ToM. More specifically, only 50% of children with ACC passed the first-order false belief task compared to 89% of the controls, and only 23% of children with ACC passed the second-order false belief task compared to 55% of the controls. The authors concluded that children with ACC between 6 and 8 years of age present a developmental delay in the ToM tasks, although the results were highly variable and not correlated with general intelligence nor with executive function. Regarding emotion and mental state recognition, children with ACC showed relatively minor difficulties in basic emotion recognition.

As pointed out by Symington et al. (2010), weaknesses in ToM could have an influence on some language usages, in particular, in the pragmatic domain. Actually, in isolated ACC, while there are no severe cognitive disabilities nor impairments in basic language skills, deficits have been found in pragmatic usages such as idioms, proverbs, and humor, and also in narratives or conversation, of growing importance in adolescence and adulthood (Lynn et al., 2003; Brown et al., 2005; Rehmel et al., 2016). Overall, less studies have been conducted with children, especially in early childhood (Siffredi et al., 2018). It must be pointed out that, in general, pragmatic usages require to go beyond the conventional meaning of words, and integrate information both from the context and the speaker’s communicative intention. The need for this integration derives from the gap between what is objectively said and what is subjectively intended by the speaker (Searle, 1993). This can also be viewed as a violation of a conversational maxim (Grice, 1989) or as a semantic conflict between words. In metaphor, especially novel metaphors, words are combined in such a way as to provoke this semantic conflict. For instance, in the metaphor “a cotton swab is a snowball,” on literal grounds, the sentence is false because a cotton swab is not snow. However, as cotton is white and soft *just as* snow, this resemblance can be transformed into a metaphorical equation. As both meanings are plausible, the listener’s task is to choose which is the one intended by the speaker. Developmental research (for a review: Winner, 1997; Melogno et al., 2012) showed that this special type of resemblance can be adequately explained even by preschoolers when the two terms of the metaphor are concrete and familiar objects, and for this reason, these metaphors are labeled as “sensory.” Some authors hypothesized that young children are able to understand sensory metaphors because they tend to create them spontaneously, although unconsciously, when they rename objects (for a review: Winner, 1997; Melogno et al., 2012) whose conventional name they already know. For instance, a preschooler may rename a basket where he put his/her foot as “boot” or rename a cookie that has the shape of the moon as “moon.” However, we must point out that production and comprehension are based on different processes. In production, what stimulates the metaphorical renaming is the perceived similarity between objects, while in comprehension, the speaker’s intentions must be reconstructed in order to harmonize the semantic conflict between words. By contrast, the figurative meaning of idioms is always conventionalized. Nevertheless, there are contrasting views about the type of processing needed to understand idioms, which can be summarized in two fundamental positions: one based on linguistic processing and the other on direct retrieval from lexicon [for a review, see Vulchanova et al. (2015)]. Differently from metaphors, idioms only have one figurative meaning, which does not change in relation to the discursive context. In addition, idioms vary as a function of various aspects. Some are ambiguous because they may have a literal as well as a non-literal meaning (e.g., “to answer the call of nature”), while others only have a figurative meaning (“to have one’s head in the clouds”). Another distinction is between decomposable and non-decomposable idioms, i.e., those cases where the meaning cannot be derived from the single constitutive words of the idiom itself. Caillès and Le Sourn-Bissaoui (2008), who investigated on the relationship between ToM and idiom comprehension in typically developing children ranging from 5 to 7, found that the performance on the ToM could only predict the performance on non-decomposable idioms. This outcome confirms the idea that ToM and figurative language comprehension are two intersecting areas where many issues concerning children with ACC are still to be clarified. Studies on both typical (Lecce et al., 2019) and atypical development, especially in individuals with autism spectrum disorders, have found a relationship between these two constructs, although the outcomes are not always convergent (Happé, 1993, 1994; Norbury, 2005; Rundblad and Annaz, 2010). For instance, in the pioneering study by Happé (1993), metaphor comprehension and success in first-order false belief task were found to be correlated. On the other hand, according to Norbury (2005), the contribution of ToM to metaphor comprehension is less crucial and would be “necessary but not sufficient.”

In addition, the study of the neural correlates of novel metaphor comprehension would confirm the relevance of a neurofunctional network where the bilateral circuits related to ToM, other circuits related to language, and further circuits related to executive function (ex: inhibition) are all activated (Menenti et al., 2009; Bambini et al., 2011; Rapp et al., 2012; Beaty et al., 2017). Interestingly, understanding a novel or a lexicalized metaphor has a different impact on brain mechanisms (Bambini et al., 2011). These issues are particularly relevant in individuals who lack, totally or partially, the most important connection between the two brain hemispheres.

A preliminary study (Melogno et al., submitted) on metaphor comprehension in a child with isolated and complete ACC revealed a notable developmental delay. Based on this outcome and the literature reviewed above, we further explored the profile of this child by adding the study of ToM and another type of figurative usage, namely idioms. We expected to find weaknesses in some ToM tasks, particularly those involving verbal language. Regarding the relationships between ToM, on the one hand, and metaphor and idiom comprehension, on the other, while we had reasons to foresee that the delay in metaphor would be associated to a weakness in the verbal ToM tasks, the outcomes regarding idioms and their relationships with ToM were less predictable.

## Materials and Methods

### Participants

Seven children participated in this study: one child (male) with isolated and complete ACC, conventionally called RJ (age: 7.2), and six controls with typical neurodevelopment. The child was recruited in NESMOS Department, “Sapienza” University of Rome. At first screening, his score on the PM47 Coloured Progressive Matrices (PM47, henceforth) (Raven et al., 1998) was at the 95th percentile. On the Similarities subtest of the Wechsler Intelligence Scale Fourth edition (WISC IV, henceforth; Wechsler, 2003), the weighted score was 10 (average range). To include children in the control group, five criteria were used: (a) age range between 7 and 7.3; (b) gender: M; (c) score on the PM47: 95th percentile; (d) weighted score at Similarities in the 9–11 range; (e) absence of sociocommunicative and learning difficulties, as reported by teachers; (f) absence of neurodevelopmental disorder; and (g) average sociocultural background, based on the study and professional level of both parents in the same city. The study was approved by the Ethics Committee of the NESMOS Department. Written informed consent was obtained from the parents for the publication of any potentially identifiable data included in this article. We will now present some basic information about RJ’s clinical history at two different stages: from birth to preschool age, and at the moment of the assessment.

### RJ’s Clinical History and Current Cognitive–Linguistic Profile

RJ was born at the 42nd week with eutocic birth. His Apgar index was 8/10, 1st and 5th min. There were no perinatal complications. His weight at birth was 4,000 g, his length was 55 cm, and his head circumference was 36.5 cm. Pregnancy had been normal with no exposure to teratogenic agents nor infections. The TORCH screening (Serum testing) was negative and fetal echocardiography was within norm. The expanded newborn metabolic screening was negative.

No syndromic picture nor nervous system pathology was revealed by family anamnesis.

Agenesis of the corpus callosum was identified with morphology ultrasound at the second pregnancy trimester, then confirmed at birth. Brain computerized axial tomography (CAT) was performed at 1 month of age and brain magnetic resonance imaging (MRI) at 2 months. No other malformation nor anatomic alteration suggestive of a genetic syndrome appeared at the clinical exam.

To exclude possible hereditary forms, a genetic study was performed on RJ and his parents, which did not highlight any significant alteration.

There was no alteration of visual or auditive functions. The electroencephalogram (EEG), repeated several times, awake and during sleep, was always within norm. No pharmacological treatment was undertaken.

Stature and ponderal growth were always within norm for the range expected for his age.

During the 1st year, parents and psychologists reported the presence of social smile, eye contact without atypicalities, shared attention, deictic gesture with declarative function at about 13–14 months, and the capability to show an object as an instrument to share with the adult.

Motor and language development were slightly delayed (first steps at 18 months and first words at 17 months). The linguistic delay has been caught up, and RJ was then able to produce both gesture-word and word associations (three words, including one verb) at about 3 years.

RJ’s cognitive profile, based on the Griffith Mental Development Scales (Luiz et al., 2006) administered at age 3 was relatively homogeneous except a weakness in the visuo-perceptual area, on the basis of which a visuo-spatial treatment was undertaken. RJ’s IQ was 95, within norm, and with a mental age of 45 months for a chronological age of 48 months, based on an assessment at age 4.4. Successive phonological, lexical–semantic, and morpho-syntactic development did not present atypicalities. Therefore, no speech therapy was undertaken.

Currently, the Intellectual child’s profile was average, based on the WISC IV (Wechsler, 2003). The IQ was 88, and was representative of the child’s intellectual ability, as well as his four indices: Verbal Comprehension Index: 86 (average); Perceptual Reasoning Index: 93 (average); Working Memory Index: 79 (below); and Speed Processing Index: 109 (average). The Working Memory Index represents a weakness from both a normative and an individual point of view.

Linguistic abilities (Marini et al., 2015), highlighted several strengths and one deficitary performance. On the production side: denomination (z: 0), semantic and phonologically fluence (z: + 1.5), sentence completion (z: −1): within norm. On the comprehension side: phonological discrimination, lexical and grammatical comprehension, Word and non-word repetition were within norm (z: 0), while Sentence repetition was deficitary (z: −2).

The average weighted scores on Children’s Communication Checklist (Bishop, 2003), completed by the parents, were speech (10), syntax (13), non-verbal communication (11), and social relationships (11). Other scores were borderline: semantics (6), consistency (6), and interests (6). Weighted score on stereotyped language was frankly in the clinical range (4). The behaviors described in item 41 of the above checklist are: “The child is too literal, sometimes with unintentional humourous outcomes.” perfectly matched the behaviors most frequently observed by the parents. Parents were asked to say how frequently their child would react like a hypothetical child, as in the following example. Ex. Hypothetical adult: “Do you find it difficult to raise in the morning?” Hypothetical child: “No, I put forward a leg from the bed, then the other one and I raise to feet.” The parents reported many similar examples of non-literal usages of language in real life where RJ remained perplexed and confused.

## Measures

### Theory of Mind

The NEPSY II (Korkman et al., 2007) includes ToM and Affect Recognition (AR). ToM includes a verbal (ToM-A) and a non-verbal contextual part (ToM-B). In the verbal part, images and small texts describing situations that require interpreting mental states, emotions, and other points of view are presented. In the contextual part, a figure showing a little girl in various social situations is presented, and the child must choose among four alternatives the face that better matches the emotional situation. Score: 0—incorrect; 1| correct. Part A Maximum score: 17; Part B Maximum score: 8. Maximum (A + B) score: 25. AR assesses the capability to recognize expressions of emotional states based on faces of children in photographs. Score: 0—incorrect; 1—correct. Maximum score: 35.

In the Pinelli and Santelli’s (2005) test, three questions, comprising two tasks each, address reality, memory, and first-order false belief. Score: 0—incorrect; 1—correct. Maximum score: 6. In the Vio and De Meo’s Theory of Mind-Central Coherence (Vio and De Meo, 2014), questions regard the character’s thought, memory, reality, and knowledge. There is also a rephrased false belief question *(“Where is the first place where A searched for x?”* Score: 0—incorrect; 1—correct. Maximum score: 6. The second-order false belief tasks (Pinelli and Santelli, 2005) include four questions, comprising two tasks each. Score: 0—incorrect; 1—correct. Maximum score: 8. The Pinelli and Santelli’s Eyes test shows two strips of moving eyes (24 stimuli) and requests to infer the corresponding emotion choosing it in a range of written alternatives. Maximum score: 24.

In the Language Assessment for Children Battery_4–12 test (It: BVL; Marini et al., 2015), the participant must infer a communicative intention (asking, stating, ordering) or an emotion (anger, happiness, sadness) from prosodic cues. Each task is multiple choice. Score: 0—incorrect; 1—correct. Maximum score: 12 for each task.

### Figurative Language

The Junior Metaphor Comprehension Test (Junior MCT; Pinto et al., 2008) assesses the capability to explain the meaning of 12 sensory metaphors in sentences, and 13 sensory metaphors in stories. Ex: “A cloud is a sponge.” The coding system is based on a three-step scale.

• Elusion: Ex: “I don’t know.” Refusal: Ex: “You can’t say this. It’s wrong.” Literal: Ex: “They gave my mum a sponge like that.” Magic: Ex: “A magician transformed the cloud into a big sponge.” Metonymical: Ex: “With the wind, the sponge flied above the cloud.” Score: 0.
• Identification of one feature common to both terms of the metaphor: Ex: “They both have the same shape.” Score: 1.
• Explanation of both similarities and differences between the two terms. Ex: “The cloud contains the rain and the sponge contains water but the sponge is much smaller and it’s in the bathroom, not in the sky.” Score: 2.

Maximum total score: 50.

Considering RJ’s weakness in the Working Memory Index and the performance on the BVL_4-12 Sentence Repetition (Marini et al., 2015), we asked him to repeat each metaphor once after the examiner had presented it and another time at the end of the response. Score: 1 point for each repetition. Total score: 50.

Idiom Comprehension (Marini et al., 2015) assesses the comprehension of 12 idiomatic sentences through multiple choice. Ex: “To lose oneself in a glass of water.” Three alternatives are given. In the above example: a) “To be unable to face a small difficulty” (correct); b) “To be unable to swim” (literal); c) “To have a very big glass of water” (metonymical). Score: 1 point for each correct answer. Maximum score: 12.

## Results

Table 1 reports RJ’s scores on the ToM tasks. These outcomes will be presented in light of the above distinction between the ToM tasks that assess visuo-perceptual cue processing and verbal ToM tasks. The performance on the Eyes test (Pinelli and Santelli, 2005) was deficitary (z: −2.86), while on the Prosodic subtests (Marini et al., 2015) and the B part of TOM in the NEPSY II were adequate. In the same battery, a relative weakness (at the border of the average range) appeared in Affect Recognition. In verbal tasks, performances were deficitary in the A part of ToM in the NEPSY II and all the false belief tasks (Pinelli and Santelli, 2005; Vio and De Meo, 2014). Nevertheless, when the standard question, “*Where must A look for the object?”* was rephrased as “*Where is the first place where A will look for x?”* (Vio and De Meo, 2014), RJ answered correctly.

**TABLE 1:** RJ’s and controls’ scores on the Theory of Mind (ToM) tasks.

	ToM A + B	ToM A	ToM B	Aff.Rec.	Fb1	Fb2	Eyes	Fb1	L.Pr.	E.Pr.
Child RJ	(13) 5	(7)	(6)	(26) 8	(2) −2.10	(2) −3.34	(12) −2.86	(2) −3.26	(5) −1/0	(12) 0/ + 2
Control 1	(18) 10	(11)	(7)	(27) 9	(6) 0.52	(8) 0.77	(20) −0.47	(6) 0.5	(7) −1/0	(11) −1/0
Control 2	(20) 12	(12)	(8)	(28) 10	(6) 0.52	(6) −0.59	(23) 0.41	(6) 0.5	(8) 0/ + 1	(12) 0/ + 2
Control 3	(19) 11	(12)	(7)	(28) 10	(6) 0.52	(7) −0.09	(21) −0.17	(6) 0.5	(6) −1/0	(12) 0/ + 2
Control 4	(20) 12	(12)	(8)	(29) 11	(6) 0.52	(6) −0.59	(21) −0.17	(6) 0.5	(8) 0/ + 1	(12) 0/ + 2
Control 5	(18) 10	(11)	(7)	(28) 10	(6) 0.52	(7) −0.09	(24) 0.71	(6) 0.5	(7) −1/0	(11) −1/0
Control 6	(17) 9	(11)	(6)	(27) 9	(6) 0.52	(6) 0.059	(20) −0.47	(6) 0.5	(5) −1/0	(12) 0/ + 2

*(From left to right) ToM A + B, Theory of Mind—part A and B; Aff.Rec, affect recognition (NEPSY II); Fb1, first-order false belief; Fb2, second-order false belief. Eyes test (Pinelli and Santelli, 2005), first-order false belief (TD-MCC); L. Pr., Linguistic prosody; E.Pr., emotion prosody (BVL_4-12). In bracket: raw scores. Out of bracket: ToM and Aff.Rec: scalar scores; Fb1, Fb2; Eyes test; Fb1, z scores; L. Pr and E. Pr, range of *z* scores.*

Table 2 reports the results on figurative language. On the Jnr MCT (Pinto et al., 2008), the total score amounted to 8, which, converted into the T score for 6-year-old children (the normative sample’s age closest to RJ’s), is equivalent to 25, a deficitary level (T ≤ 30), while the controls ranged from average to superior. In terms of quality of the responses, the dominant level was the lowest (0 score: 68%). Among these 0 level responses, refusals were 64.71%, followed by metonymical (23.53%) and literal responses (11.76%).

**TABLE 2:** RJ’s scores and controls’ on metaphor comprehension (Jnr MCT) and idiom comprehension (BVL_4-12).

	Jnr MCT				BVL_4-12
	Rep	Dec Met	Con Met	Tot Met	Idiom
Child RJ	(50)	(2)	(6)	(8) T:25	(2) z:−1.5/−1
Control 1	(50)	(11)	(13)	(24) T: 54	(5) z: 0/ + 1
Control 2	(50)	(13)	(13)	(26) T: 58	(3) z: −1/0
Control 3	(50)	(16)	(17)	(33) T: 71	(5) z: 0/ + 1
Control 4	(50)	(14)	(15)	(29) T:63	(5) z: 0/ + 1
Control 5	(50)	(13)	(18)	(31) T: 67	(3) z: −1/0
Control 6	(50)	(18)	(18)	(36) T: 76	(4) z: −1/0

*Rep, item repetition; Dec. Met, decontextualized metaphors; Con. Met, contextualized metaphors; Tot Met, Total test; Idiom, Idiom comprehension. In bracket: raw scores. T, T scores for tot Jnr MCT; Idiom, range of z scores.*

On the BVL_ 4-12 (Marini et al., 2015) Idiom Comprehension subtest, the performance was borderline (z between − 1.5 and −1).

To compare RJ’s performance to the controls’, we applied the Crawford et al. (2010) method that allows to compare an individual to control groups with modest *N* (e.g.,<10). The statistics of the control group are then treated as sample statistics rather than population parameters, and the *t*-distribution (with N–1 degrees of freedom) is used, rather than the standard normal distribution, to evaluate the abnormality of the individual’s scores. Given the different kind of transformed scores provided by the tests administered, the comparisons were performed on raw scores. The *t*-test results are presented in Table 3, in which the estimated percentages of normal population falling below RJ’s score (Estimate%), the effect sizes (z_*cc*_), and the lower and upper limits of a 95% confidence interval for the true effect size are also reported.

**TABLE 3:** RJ’s performance compared to the controls’.

Measures	Control group mean	Control group SD	Child RJ’s score	t Test	p (two-tailed)	Estimate% *	Effect size (z_*cc*_)	Lower limit **	Upper limit **
ToM (A + B)	18.67	1.11	13.00	−4.73	0.0050	0.26%	−5.11	−8.27	−1.96
ToM A	11.50	0.50	7.00	−8.33	0.0004	0.02%	−9.00	−14.47	−3.60
ToM B	7.17	0.69	6.00	−1.57	0.1772	8.86%	−1.70	−2.96	−0.37
Aff.Rec.	27.83	0.69	26.00	−2.46	0.0580	2.88%	−2.65	−4.42	−0.86
FbII	6.67	0.75	2.00	−5.77	0.0020	0.11%	−6.23	−10.05	−2.44
Eyes	21.50	1.50	12.00	−5.86	0.0020	0.10%	−6.33	−10.22	−2.48
L.Pr.	6.83	1.07	5.00	−1.58	0.1740	8.71%	−1.71	−2.99	−0.38
E.Pr	11.67	0.47	12.00	0.65	0.5440	72.78%	0.70	−0.23	1.58
Dec. Met	14.17	2.27	2.00	−4.96	0.0040	0.21%	−5.36	−8.67	−2.07
Con. Met	15.67	2.13	6.00	−4.20	0.0080	0.42%	−4.54	−7.37	−2.07
Tot.Met.	29.83	4.06	8.00	−4.98	0.0040	0.21%	−5.38	−8.70	−2.07
Idiom	4.17	0.90	2.00	−2.23	0.0760	3.80%	−2.41	−4.05	−0.74

ToM. A + B, Theory of Mind—part A and B (NEPSY II); Aff. Rec, Affect recognition (NEPSY II); Fb2, second-order false belief (Pinelli and Santelli, 2005); Eyes Test (Pinelli and Santelli, 2005). L. Pr., linguistic prosdy; E.Pr., emotion prosody (BVL_4–12); Dec. Met, decontextualized metaphors; Con. Met, contextualized metaphors; Tot Met, Jnr. MCT; Idiom, idiom comprehension.*Estimated percentage of normal population falling below RJ’s score.**Lower and upper limits of a 95% confidence interval for the true effect size.N.B. The comparisons on Fb1 (Pinelli and Santelli, 2005) and Fb1 (Vio and De Meo, 2014) and Repetition were not performed because the SD was 0 in the control group.

Differences were significant in favor of the controls for the three measures of the Jnr MCT (Dec. Met: *t* = −4.96, *p* = 0.004, z_*cc*_ = −5.36; Con.Met: *t* = −4.20, *p* = 0.008, z_*cc*_ = −4.54; Tot. Met.: *t* = −4.98, *p* = 0.004, z_*cc*_ = −5.38), ToM total score (*t* = −4.73, *p* = 0.005, z_*cc*_ = −5.11), ToM A score (*t* = −8.33, *p* = 0.0004, z_*cc*_ = −9.00), second-order false belief task (*t* = −5.77, *p* = 0.002, z_*cc*_ = −6.23), and Eyes test (*t* = −5.86, *p* = 0.002, z_*cc*_ = −6.33). Affect recognition and Idiom Comprehension only approached significance, and no difference appeared in Prosodic cues, Linguistic and Emotional cues nor in ToM-B.

## Discussion

In this article, we studied ToM and figurative language comprehension in a 7.2-year-old child, RJ, with isolated and complete AC, two particularly challenging abilities in this condition. At preschool age, his basic abilities appeared adequate. Some weakness had been noticed since the beginning of primary school in both the understanding of non-literal usages and everyday communicative exchanges. Phonological, lexical, and grammatical abilities were adequate for his age in both production and comprehension. At the time of the present study, RJ’s overall intellectual level was adequate in spite of a poor performance in working memory. Regarding ToM, the outcomes were highly diversified in relation to the nature of the stimuli, visual and perceptual, on the one hand, and verbal, on the other. Theory of Mind (Part B) and Affect recognition were adequate when the stimuli were pictures of children’s faces (Korkman et al., 2007), and deficitary in the Eyes test (Pinelli and Santelli, 2005). On the contrary, emotions and communicative intentions were adequately recognized from prosodic cues (Marini et al., 2015), in line with some results of the Labadi and Beke’s (2017) study where no deficit in basic emotion recognition was found in children with ACC in the 6- to 8-year range. RJ failed the first-order false belief task (Pinelli and Santelli, 2005), normally solved at about 4 years, and this delay was confirmed when compared to a control group. Nevertheless, when the usual direction of the task *(“Where will A look for x?”)* was rephrased as “*Where is the first place where A will look for x?* (Vio and De Meo, 2014), RJ responded correctly. The contrast between the two performances could be due to the difficulties the child experienced when he had to grasp the communicative intention of the examiner. According to Siegal and Beattie, 1991; Siegal and Peterson, 1994), the failure on the false belief task may be due to a violation, on the part of the examiner, of the Gricean quantity maxim (Grice, 1989), and the child would be unable to detect. Overall, RJ’s performances on the NEPSY II ToM tasks (Part A) (Korkman et al., 2007), as well as on the Pinelli and Santelli’s (2005) second-order false belief task, were under average. In both cases, these tasks involve more complex reasoning, and individuals with ACC generally fail, as pointed out by Brown and Paul (2019).

Regarding figurative language, RJ’s performance on metaphors was deficitary, again with a delay of 3 years, once more confirmed when compared to the controls. In the majority of responses, the level was the lowest. RJ’s performance on idioms was at the border of average level. However, this task is a multiple-choice one. When RJ was asked to justify his responses, he would explain them on literal grounds in either correct and incorrect cases. For example, to explain the idiom “to look for a needle in a haystack,” where he performed well, he said: “the cows in a haystack… there are cows that hide a needle and then look for it.” In the Jnr MCT (Pinto et al., 2008), the prevalence of refusals (e.g., for the item “The moon is a light bulb”: “It’s absolutely nonsense, the moon is not a light bulb.”) suggests that the literally false meaning of the utterance was not inhibited in favor of a plausible metaphorical meaning.

RJ’s profile presents several discrepancies. These could be accounted by a general difficulty in inferring communicative intentions, which appeared both when RJ had to interpret the classical verbal directions of the false belief tasks and when he had to interpret the communicative intention underlying the non-literal meaning in metaphors. This type of explanation follows a well-known interpretative line, inaugurated by the early Happé’s (1993) studies. The low performance on the Jnr MCT (Pinto et al., 2008) could be due to RJ’s unawareness that the speaker does not really mean what he/she says. On the other hand, to come to a plausible interpretation of a metaphor, one must also detect some similarities between the two terms that constitute it, like in the above “cotton/snow” example. While this capability to detect similarities did not appear in Jnr MCT’ performance, it did so in the WISC IV Similarities (Wechsler, 2003), which was average. This discrepancy could be explained by the fact that, in Similarities, the task requests to identify semantic features in words belonging to the same category (ex: “apple” vs “banana”), while in metaphor, words belong to heterogeneous categories (ex: “cloud” vs “sponge”). In addition, while in Similarities the two terms are simply juxtaposed, in a metaphorical sentence, they are united by the verb “to be,” which renders that sentence implausible on literal grounds. Surprisingly, in both sensory metaphor comprehension and the Fb1 tasks, RJ showed a 3-year delay. In metaphor comprehension and the Pinelli and Santelli’s false belief task Pinelli and Santelli’s (2005), there was an *implicit* request to fill up the gap between what is literally said and what is subjectively intended (Grice, 1989; Searle, 1993). By contrast, when this request was made *explicit*, RJ could solve the problem just as he was able to find resemblances where these were explicitly demanded in Similarities. Similarly, some studies (Melogno et al., 2017a,b) showed that children with ASD who also tended to refuse metaphors, accepted to search for the relevant similarities between the two terms of a metaphor when an adult suggested to insert “is like” between these terms, making the linkage explicit. Therefore, in RJ’s linguistic profile, the weakness seems to lie in the pragmatic rather than in the semantic dimension.

We must also point out that metaphor comprehension requires a heavier working memory load to find a resemblance than in Similarities. Detecting common features and, at the same time, inhibiting the irrelevant ones, updating information and shifting flexibly from one term of the metaphor to the other, all these executive functions (Miyake et al., 2000) overload working memory, which is precisely a weak point in RJ’s profile. Indeed, the study of the various components of ToM and figurative language are particularly relevant in individuals with ACC. For instance, in novel metaphor comprehension (Bambini et al., 2011), as well as in production (Beaty et al., 2017) but also in those ToM tasks that require to integrate perceptual and textual cues, the absence of the corpus callosum raise a problem with respect to the activation of bilateral networks. To date, two main interpretations have been proposed: the laterality interpretation, according to which the information, better understood by a hemisphere, would not be available to the other one, and the reduction in the size of functional networks that would limit the processing, especially of multisensory information (Symington et al., 2010). Nevertheless, in children as young as RJ, we must also consider that that the brain is still in development.

We are aware that this study presents some limitations. Among these, we will point out that we could have better explored the difference between metaphor and idiom comprehension by assessing each of these figures with two types of instruments, one based on multiple choice, and the other on explanation. This would have allowed us to distinguish between the specific constraints linked to the very nature of each figure and those linked to the assessment modality. Overall, RJ does not meet the classification criteria for ASD, and his difficulties seem to be circumscribed to the pragmatic domain. Actually, RJ never presented that early onset of repetitive and stereotyped behaviors, which apparently characterized the individuals with overlapping between ASD and ACC described by Paul et al. (2014).

We believe this report offers an original approach to the exploration of higher-order cognitive processes such as ToM, sensory metaphors, and idioms in young children with complete ACC. In addition, as far as we know, this paper fills a gap in the literature on metaphor comprehension in children with isolated and complete ACC at such an early age as RJ’s. Novel sensory metaphors could be an early indicator of pragmatic difficulties in this population or in a subgroup of this population. The study of the relationships between ToM and figurative language in children with ACC could contribute to a better definition of their specific difficulties in the pragmatic domain. Based on the future outcomes of this line of research, but also exploiting already implemented interventions on children with ASD (Melogno et al., 2017a,b) showing weaknesses in non-literal comprehension similar to RJ’s, treatments could be conceived to enhance pragmatic linguistic usages in children with ACC.

## Data Availability Statement

The raw data supporting the conclusions of this article will be made available by the authors, without undue reservation.

## Ethics Statement

The studies involving human participants were reviewed and approved by Sant’Andrea Hospital Sapienza University Rome. Written informed consent to participate in this study was provided by the participants’ legal guardian/next of kin.

## Author Contributions

SM was responsible for the research design, was one of the authors of one of the tests, coordinated the team and supervised the whole research. MAP was one of the authors of one of the tests, and supervised the whole writing process. TS supervised the statistical methodology. FB contributed to the data collection. PP was responsible for the data. All authors contributed to the article and approved the submitted version.

## Conflict of Interest

The authors declare that the research was conducted in the absence of any commercial or financial relationships that could be construed as a potential conflict of interest.
